# Characteristics of Gut Microbiota and Fecal Metabolites in Patients with Colorectal Cancer-Associated Iron Deficiency Anemia

**DOI:** 10.3390/microorganisms12071319

**Published:** 2024-06-28

**Authors:** Qinyuan Zhang, Wen Wu, Fanying Guo, Jinming Li, Yutao Jin, Guoxiang Cai, Yongzhi Yang

**Affiliations:** 1 Department of Colorectal Surgery, Fudan University Shanghai Cancer Center, Shanghai 200032, China; 2 Department of Oncology, Shanghai Medical College, Fudan University, Shanghai 200032, China

**Keywords:** colorectal cancer, gut microbiota, gut metabolites, iron deficiency anemia

## Abstract

Patients with colorectal cancer (CRC) have a high prevalence of iron deficiency anemia (IDA), and the gut microbiota is closely related to iron metabolism. We performed metagenomic and metabolomic analyses of stool samples from 558 eligible samples, including IDA CRC patients (IDA, n = 69), non-anemia CRC patients (Non-Anemia, n = 245), and healthy controls (CTRL, n = 244), to explore the dynamically altered gut microbes and their metabolites. Compared with the CTRL group, fecal bacteria in both the IDA group and the Non-Anemia group showed a decrease in alpha diversity and changes in microbial communities. *Flavonifractor plautii* (*F. plautii*) increases progressively from CTRL to Non-Anemia to IDA, accompanied by decreased trimethoxyflavanone and a downregulated KO gene, megDIII. In the Non-Anemia group, *Parabacteroides* showed a specifically elevated abundance positively correlated with enriched 1,25-dihydroxyvitamin D3. The intricate correlations among gut microbiota, metabolites, and KO genes were uncovered and highlighted, implicating an aberrant iron metabolism vulnerable to chronic inflammation during the deterioration of the anemic condition. Furthermore, the amount of *F. plautii* in feces achieved independent and effective prediction performance for the poor outcome of CRC. Perturbed host-microbe interplays represent a novel prospect for explaining the pathogenesis of CRC-associated IDA. The fecal microbial features also reflect the associations between IDA and elevated CRC recurrence risk.

## 1. Introduction

Prior statistics have estimated that the morbidity of cancer-related anemia (CRA) ranges from 30 to 75.8% in the context of colorectal cancer (CRC) [1]. The etiologies of CRA vary, including myelosuppressive chemotherapy, tumor-associated bleeding, chronic inflammation, drug-induced erythrocyte destruction, etc. [2]. Iron deficiency anemia (IDA), which accounts for most of CRA [3,4], is a common comorbidity in patients with CRC. It also acts as an independent risk factor responsible for adverse postoperative outcomes [5,6]. The pathogenesis of IDA in CRC is complicated due to multiple physiological factors and individualized treatments. Anatomical and clinical features, including the right colon, tumor stage, hypoalbuminemia, and positive fecal occult blood test (FOBT), were recognized as hazardous indicators for forecasting the onset and advancement of IDA in CRC patients [7]. Ferritin is an indicator of iron status applied in practice [8]; however, it cannot reliably quantify iron stores in inflammatory circumstances (such as cancer) as an acute-phase reactant [9]. Meanwhile, considering the unpleasant side effects presented by intravenous iron or red cell transfusion, including increased infection rates, exacerbated tumor progression, and higher mortality, fewer CRC patients were in a reasonable anemia regime [10,11].

Several significantly and potentially altered IDA-related microbiota have been identified by microbial sequence-based profiling and functional analysis [12]. Iron plays a vital role for the microbiota since it participates in a spectrum of metabolic enzymatic reactions as a cofactor, supporting bacterial growth, colonization, and virulence [13]. Microbial communities have evolved with high-affinity iron-chelating siderophores to compete for luminal iron, indicating their sensitivity to gut iron status [14]. Meanwhile, gut microbiota also affect the homeostasis of iron in the host. The addition of single species (such as *Bacteroides thetaiotamicron*, *Faecalibacterium prausnitzii*, and the probiotic *Streptococcus thermophilus*) into germ-free animals has been observed to be capable of enhancing the retention of body iron by upregulating the expression of intestinal iron transporters Dcytb, Dmt1, and hephaestin [15]. Likewise, metabolites derived from the microbiota are relevant to iron disorders. For instance, *Lactobacillus* species inhibit host iron absorption by detecting the level of intestinal iron and producing 1,3-diaminopropane (DAP) and reuterin, both of which are inhibitors of hypoxia-inducible factor (HIF-2α), suppressing intestinal HIF-2α activity in iron absorption and storage [16].

Currently, no study has reported the association between gut microbiota and IDA associated with CRC. In this study, we initiated large-sample dual-omics analyses to picture the microbial characteristics of IDA and the non-anemic condition, respectively, in the CRC setting. These markers, after stringent selections, further shed light on a novel direction to assess iron deficiency in CRC patients. Taken together, we demonstrated a new diversion devoted to the potential mechanisms underlying the correlations among IDA-featured gut microbiome and metabolome, IDA, and recurrence-free life expectancy.

## 2. Materials and Methods

### 2.1. Participant Information and Fecal Collection

**Figure 1:**
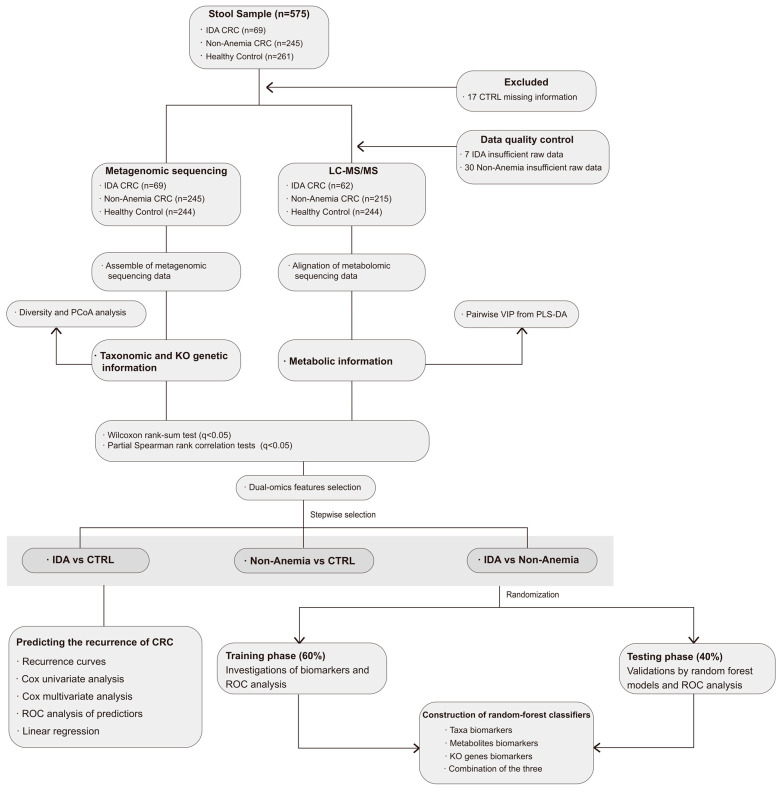
Flowchart of the study. The metagenomic and metabolomic sequencing was performed on fecal samples from recruited participants, and further analyses were conducted to obtain the exceptional microbial and metabolic signatures of the IDA CRC and Non-Anemia CRC groups. The associations among the multi-feathers were identified. Random-forest classifiers were built based on the signatures.

A total of 575 fecal samples from 69 IDA CRC patients (IDA group), 245 non-anemia CRC patients (Non-Anemia group), and 261 healthy controls (CTRL group) were collected prospectively at Fudan University Shanghai Cancer Center (Figure 1). All CRC patients were newly diagnosed according to a colonoscopic pathological biopsy, among which patients with a family history of CRC, irritable bowel syndrome, and other coexisting malignancies were excluded. The information on tumor size, infiltrating nodes, and metastasis was collected to grade the tumor stage. Synchronously with the diagnosis of CRC, IDA was diagnosed before any tumor or anemic treatment in the cohort. Based on the published algorithm to predict response to iron in cancer-associated anemia [2], CRC-related IDA (including absolute iron deficiency anemia (AIDA) and functional iron deficiency anemia (FIDA)) was diagnosed if it met the following criteria: (1) A decrease in hemoglobin (Hb) with Hb < 120 g/L in males or <110 g/L in females. Red cell morphology presented in microcytic hypochromic form. (2) The classic manifestations of iron deficiency (such as fatigue, dizziness, palpitations, etc.). (3) Transferrin saturation < 20% and serum ferritin < 500 ng/mL. CTRLs were confirmed free from gastrointestinal tumors and anemia via colonoscopic screening and hematological examinations, respectively. All included volunteers or patients were not using antimicrobials or probiotics within 1 month prior to fecal sampling. Patients who had been treated with any types of anticoagulant therapies (including low-molecular-weight heparin, vitamin K antagonists, and direct oral anticoagulants) within 1 month were excluded from this study. This study was conducted in compliance with the 1964 Helsinki Declaration and was approved by the Institutional Review Board of Fudan University Shanghai Cancer Center. The feces were collected before preoperative bowel preparation and stored at −80 °C, refraining from degeneration before extraction.

### 2.2. Fecal DNA Extraction, Library Construction, and Metagenomic Sequencing

Fecal DNA was extracted using the QIAamp DNA Stool Mini Kit (Qiagen, Hilden, Germany). DNA concentrations were measured by NanoDrop spectrophotometry (NanoDrop, Paqlab, Germany), while DNA quality was examined by agarose gel electrophoresis. Sequencing libraries were constructed using the TruSeq Nano DNA LT Library Preparation Kit (Illumina, San Diego, CA, USA) according to the manufacturer’s specifications, as previously described [17]. The prepared products were applied to the NovaSeq6000 platform (Illumina). Raw sequencing reads were processed to obtain valid reads and assembled using IDBA-UD (V.1.1.1). After being compared to the database (V.202003, (ftp://ftp.ccb.jhu.edu/pub/data/kraken2_dbs/ (accessed on 20 June 2022))) using Kraken2 software (V.2.1.1) and Braken software (V.2.5), species-level information was harvested. The α diversity (Chao1 index) and β diversity (Bray-Curtis distance) were calculated using the vegan package in R (V.3.6.3). Principle coordinate analysis (PCoA) was performed on the sample pairwise. For continuous variable comparison, a two-tailed Wilcoxon rank-sum test was used. A multivariate association with linear models (MaAsLin) framework was prepared for correcting confounding factors (age and sex) in taxonomic analyses. Moreover, the abundance of KO genes (Kyoto Encyclopedia of Genes) and the related KEGG (Kyoto Encyclopedia of Genes and Genomes) pathway in HUMAnN3 were found. Difference analyses on the KO gene profile were performed among three groups using a two-sided Wilcoxon rank-sum test to define significance with an adjusted *p*-value (q value) < 0.05 by the Benjamini–Hochberg procedure. Lists of KO genes were obtained from KEGG BRITE ‘ko00001.keg’ with the keywords ‘Environmental Information Processing’, ‘Metabolism’, and ‘Cellular Processes’.

### 2.3. Fecal Metabolite Extraction and Liquid Chromatograph Mass Spectrometry (LC-MS) Analysis

The procedure for qualified fecal metabolite preparation was strictly based on the supporting information [18]. The ultra-performance liquid chromatography system (SCIEX, Framingham, MA, USA) was operated, and the eluted metabolites were detected by the high-resolution tandem mass spectrometer TripleTOF5600plus (SCIEX, Framingham, MA, USA). The pretreatments were settled for acquiring MS data using the XCMS software version 3.5.1. After a series of processes, the raw LC-MS data were aligned to the matched exact molecular mass data (*m*/*z*) in the online KEGG and HMDB databases and finally underwent quality control according to the published protocol [19]. Principal component analyses were executed to filter out samples outside the 95% confidence interval prior to fecal metabolome analyses. Only confidently annotated metabolites were selected for differential abundance analyses. Differentially altered metabolites were required to meet the following standards: (1) variable importance for the projection (VIP) > 1 using partial least squares discriminant analysis (PLS-DA), (2) log2-transformed fold change (FC) of >1.5 or <0.67, and (3) *p* value after tailed area-based false discovery rate correction (q value) < 0.05.

Procrustes analyses were deployed to test the associations among altered taxa (mean relative abundance > 1%), metabolites, and KO genes (both mean relative abundances > 10%) (Table S1). Subsequently, the partial Spearman rank correlation test (PResiduals package version 0.2-5) was used to search for the correlations between any two pairs of taxa/metabolites/KO genes among all samples in the IDA, Non-Anemia, and CTRL groups.

### 2.4. The Correlations between Microbial Features and Cancer Recurrence

Survival analysis was conducted using Kaplan–Meier estimates, and we estimated the differences between the two recurrent curves using the log-rank test. Next, subgroup analyses on *F. plautii* abundance were performed in our study according to anemic condition and recurrence. Cox univariate and multivariate analyses (survival package version 3.2-10 and survminer package version 0.4.9) were required to reveal the predictive risk factors for RFS (recurrence-free survival). ROC curve analysis was conducted for the prediction of CRC recurrence using either *F. plautii* abundance or AJCC stage. The linear correlation analysis was performed to expose the association between RFS and *F. plautii* abundance.

### 2.5. Random Forest and Biomarker Identification

A random forest was built to validate the efficacy of classification models based on species, metabolites, KO genes, and a combination of the three. For input features, classifiers incorporated all significantly altered taxa, metabolites, or KO genes. First, a feature importance score (the mean decrease in accuracy, which designates the feature contributing to the accuracy of the model) was calculated in the random forest algorithm. Second, the cross-validation error curve obtained by using the tenfold cross-validation method assisted in determining the best formulation of features in taxa and metabolites, and the fivefold cross-validation method was used for the selection of KO markers. The ROC curve was constructed (pROC package version 1.12.1) to evaluate the models, and the AUC was used to exhibit the ROC effects.

### 2.6. Statistical Analysis

Continuous variables displayed as the mean ± standard deviation were applied to a two-sided Wilcoxon rank-sum test, while categorical variables presented as numbers (percentages) were appropriate for Pearson’s χ^2^ test or Fisher’s exact test. Spearman rank correlation analyses were required to identify the correlations among microbes, metabolites, and KO genes. Statistical analyses depended on GraphPad Prism V.8.0 software (GraphPad Software, San Diego, CA, USA), R V.3.6.3 (R Foundation for Statistical Computing, Vienna, Austria) and Microsoft Office Excel 2013 (Microsoft, Seattle, WA, USA).

## 3. Results

### 3.1. Patient Characteristics

Of the 575 enrolled volunteers, 17 volunteers in the CTRL group were excluded because of missing information. Among the 558 eligible volunteers, 69 CRC patients were diagnosed with IDA, 245 CRC patients were not anemic, and 244 were healthy controls (Figure 1). Demographic characteristics such as age, gender, and body mass index were well matched among the three groups. The clinical pathological characteristics of CRC (including TNM staging, non-quantitative FOBT, nerve invasion, and vascular invasion) as well as tumor markers (serum CEA) were not considerably different between the two disease groups. IDA was more likely to occur in patients with a larger tumor size (tumor size ≥ 5 cm: 37.7% vs. 23.7%) or in the right hemicolon (tumor location in the right hemicolon: 49.3% vs. 13.9%). Body nutritional indicators, including albumin and prealbumin, were lower in patients with IDA (albumin < 40 g/L: 31.9% vs. 8.6%; prealbumin < 280 mg/L: 88.4% vs. 62.0%). Compared with the Non-Anemia group, the level of hemoglobin (Hb) (98.68 ± 18.76 g/L vs. 145.32 ± 15.33 g/L in males; 88.45 ± 15.67 g/L vs. 131.69 ± 14.42 g/L in females) remarkably decreased in the IDA group. Consistently, the hematocrit showed a similar tendency (32.04 ± 5.12% vs. 43.23 ± 2.72% in males; 29.20 ± 4.44% vs. 39.49 ± 2.67% in females). The detailed clinical data are shown in Table 1.

### 3.2. The Gut Microflora Dysbiosis

A significantly decreased α diversity index measured by the Chao1 Index (*p* = 0.021 and 0.00011, respectively, Wilcoxon rank-sum test) (Figure S1A) was observed in both the IDA and Non-Anemia groups. Principal coordinate analyses (PCoA) were conducted based on the distributions of the features (PERMANOVA test, IDA vs. CTRL: pseudo-F: 9.22, *p* = 0.01; Non-Anemia vs. CTRL: pseudo-F: 21.25, *p* = 0.001; IDA vs. Non-Anemia: pseudo-F: 1.11, *p* = 0.34) (Figure S1B). In both disease groups, the overall microbiome compositions were distinguished from those of the CTRL group. However, the bacterial diversity and composition of the IDA group failed to be differentiated from the Non-Anemia group.

### 3.3. Taxonomic Signatures of the IDA Group and Non-Anemia Group

**Figure 2:**
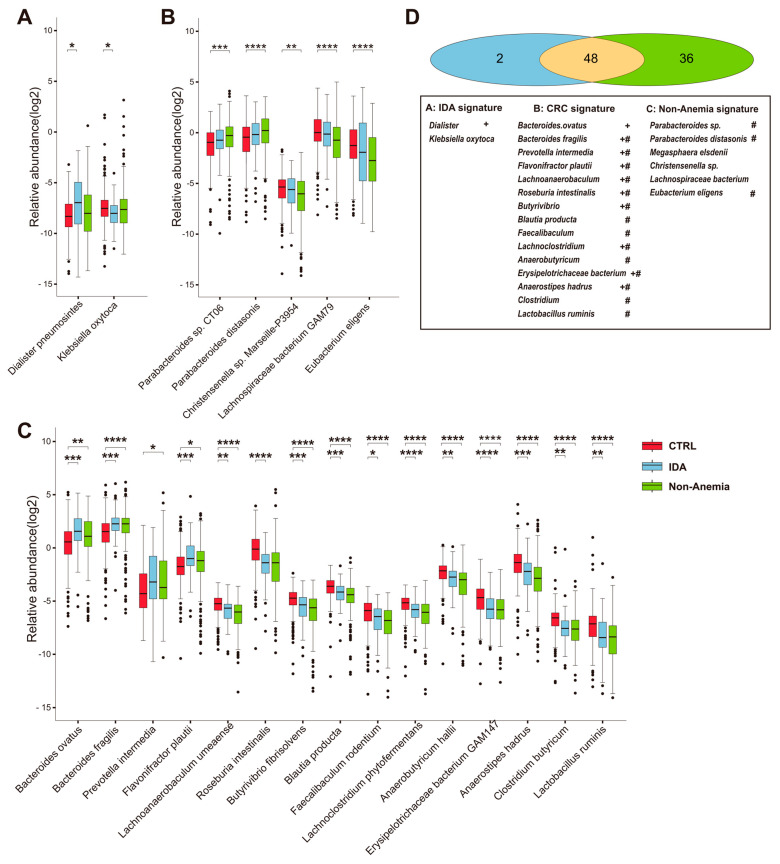
A broad review of the species-level taxonomic distribution of the gut microbiome. Boxplots show the relative abundance of species altered in the (**A**) IDA group, (**B**) Non-Anemia group, and (**C**) both disease groups. (**D**) Venn diagram outlining the taxa signatures that significantly changed in one disease status (q < 0.05, Wilcoxon rank-sum test) but not in the other (q > 0.2, Wilcoxon rank-sum test). Significant changes (elevation and depletion) are denoted as follows: * q < 0.05, ** q < 0.01, *** q < 0.001, **** q < 0.0001. + and # denote species associated with the IDA group and the Non-Anemia group, respectively, after using MaAsLin (version 1.8.0) adjusting for age and gender.

The snapshots of individual species in the IDA and Non-Anemia groups were provided by comparing the relative abundances between (1) IDA and CTRL, (2) Non-Anemia and CTRL, and (3) IDA and Non-Anemia. We further exhibited taxonomic signatures that significantly changed in one disease status (q < 0.05, Wilcoxon rank-sum test) but not in the other (q > 0.2, Wilcoxon rank-sum test) compared with the CTRL group. A total of 50 species with differential abundances between the IDA group and the CTRL group were shown (q < 0.05, Wilcoxon rank-sum test; Table S1). Multivariate association with linear models (MaAsLin2) was then applied to control the potential confounding factors (including age and gender), and 40 out of 50 species still reached statistically significant positions (q < 0.05, MaAsLin; Table S1). Notably, only the increased *Dialister pneumosintes* was specific to the IDA group (Figure 2A,D). In the Non-Anemia group, the relative abundances of 84 species were different from the CTRL group (q < 0.05, Wilcoxon rank-sum test; Table S2), 74 of which withstood correction for confounders (q < 0.05, MaAsLin; Table S2). A total of 36 taxa were exclusively altered, among which *Parabacteroides* sp. *CT06* and *Parabacteroides distasonis* were overrepresented, while *Eubacterium eligens* and *Lachnospiraceae bacterium GAM79* were underrepresented (Figure 2B,D). Notably, fewer differences were observed between the IDA group and the Non-Anemia group, with 16 species showing suggestive differences (*p* < 0.05, Wilcoxon rank-sum test). Levels of *Flavonifractor plautii (F. plautii)* and *Bacteroides thetaiotaomicron* tended to increase as iron deficiency worsened, but remained non-significantly different after adjustment for multiple covariates (Table S3).

Compared with the CTRL group, the IDA and Non-Anemia groups demonstrated substantial overlap in the changes in the individual taxa. In total, 48 taxa were associated with both CRC groups (q < 0.05, Wilcoxon rank-sum test; Tables S1 and S2), including increased *Bacteroides fragilis*, *Bacteroides ovatus*, and *Prevotella intermedia*, as well as decreased *Faecalibacterium prausnitzii*, *Eubacterium rectale*, and *Christensenella minuta* (Figure 2C,D).

In brief, these results illustrated the distinct and common characteristics of the gut microbiome at the species level. These findings might be linked with variations in the clinicopathological traits involved in iron deficiency.

### 3.4. Fecal Metabolomic Alterations in the IDA Group and the Non-Anemia Group

**Figure 3:**
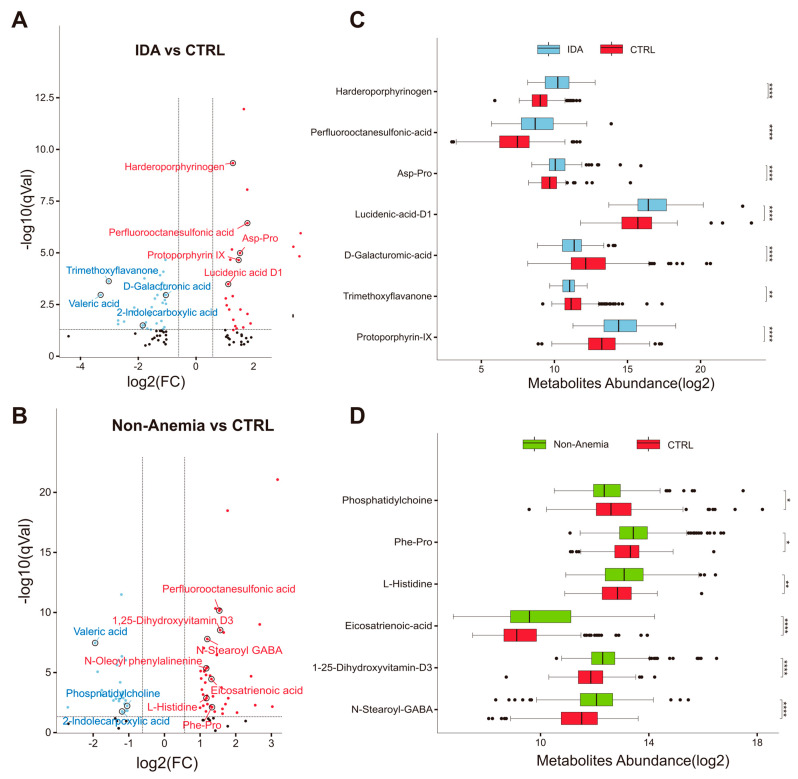
Fecal metabolome changes in the disease groups versus the CTRL group. Volcano plots demonstrating metabolite changes in the (**A**) IDA group and the (**B**) Non-Anemia group compared with the CTRL group, respectively. The X axis shows log2-transformed FC of fecal metabolite abundances, and the Y axis represents the log10-transformed q value analyzed using the Wilcoxon rank-sum test. The horizontal lines represent q < 0.05, and the vertical lines indicate an FC of >1.5 or <0.67. Metabolites that increased or decreased are highlighted in red or blue, respectively. (**C**,**D**) Boxplots showing representative metabolites that were significantly changed in the IDA or Non-Anemia groups after controlling confounding factors. * q < 0.05, ** q < 0.01, **** q < 0.0001.

Given the close interactions between gut microbiota and the host, we employed untargeted metabolomics on fecal samples (IDA: n = 62; Non-Anemia: n = 215; and CTRL: n = 244) to reveal discrepant metabolites that might be the driving force towards IDA. OPLS-DA showed no significant difference in metabolic composition between the IDA group and the Non-Anemia group, but both were distinct from the CTRL group, which is consistent with the variations in data from the fecal microbiome described above (Figure S2A). Next, we demonstrated the relevant abundance of each annotated metabolite featured in the IDA or Non-Anemia groups. Accordingly, 26 metabolites were exclusively changed in the IDA group (Table S4), including the depletion of potential anti-inflammatory plant polyphenols such as trimethoxyflavanone [20], austroinulin [21], and quinic acid [22]. Alongside these, protoporphyrin IX and prostaglandin H2 were enriched (Figure 3A,C and Figure S3). A total of 33 metabolites were identified as merely altered in the Non-Anemia group (Table S5), among which the potential anti-inflammatory mediators such as 1,25-dihydroxyvitamin D3 [23] and eicosatrienoic acid [24] were dramatically increased. In addition, several amino acid metabolites that have been reported to facilitate erythropoiesis, including acetyl-DL-valine [25], pristanoylglycine [26], and phenylalanine [27,28], were significantly increased solely in the Non-Anemia samples (Figure 3B,D and Figure S3).

Furthermore, both CRC groups shared overlapping characteristics, with 24 metabolites concordantly altered compared with the CTRL group (Tables S4 and S5). The ecotoxic metabolite perfluorooctanesulfonic acid was remarkably increased. In addition, 2-indolecarboxylic acid (metabolite derivatives of tryptophan) and valeric acid belonging to the group of short-chain fatty acids (SCFAs) were downregulated (Figure S3). When compared with the Non-Anemia group, there was an upward trend on lucidenic acid D1 and a downward trend on austroinulin in the IDA group, yet neither of which showed a significant difference after false discovery rate correction (q > 0.05; Table S6). The noticeably altered compounds specific to each group need to be further studied to determine potential microbe–metabolite interactions.

### 3.5. Changes in Microbial Genes Summarized in KO Genes and KEGG Pathway Modules

To profile alterations in the functional level of microbiomics, including relative gene abundances and their corresponding pathways owing to microbial shifts, we aligned metagenomic DNA sequences to the UniRef90 database and utilized the HUMAnN3 tiered search strategy to quantify the KEGG Orthologous (KO) term abundance in the KEGG database. In the PCoA, noticeable differences in the distribution of KO genes were spotted only between the IDA and CTRL groups and between the Non-Anemia and CTRL groups (Figure S2B). Predominant KOs were identified by the Wilcoxon rank-sum test. We then annotated these differential KOs, which were enriched in specific pathways referred to as Environmental Information Processing (ko00001.keg), Metabolism (ko00001.keg), and Cellular Processes (ko00001.keg).

**Figure 4:**
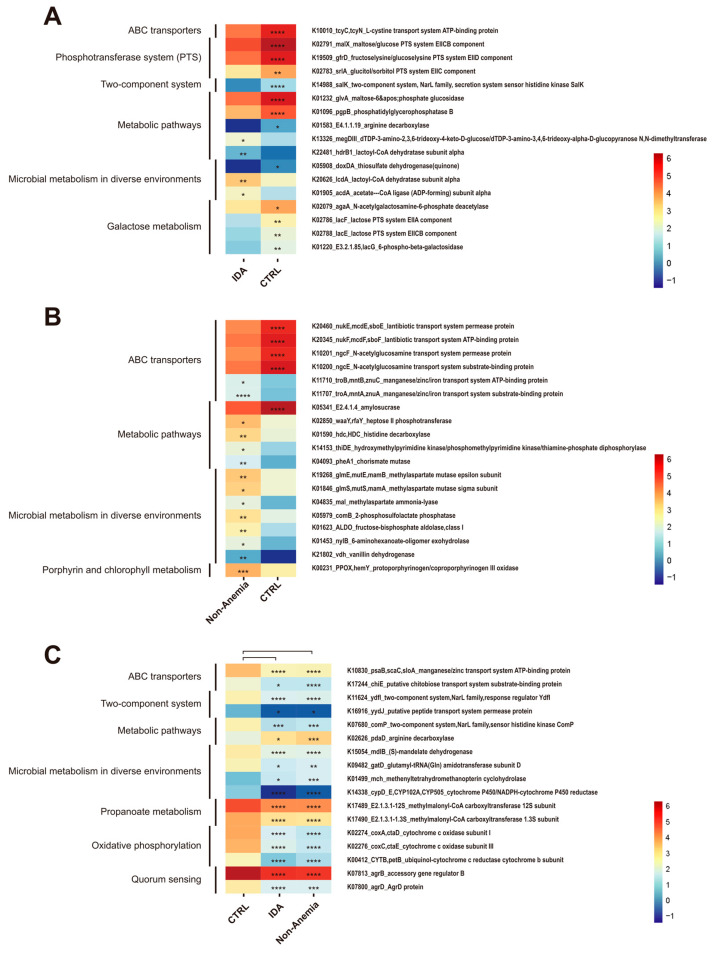
Gut microbiome functionality profiles. Functional pathways with enriched KOs were identified by the Wilcoxon rank-sum test (q < 0.05) between the (**A**) IDA group and the CTRL group, and the (**B**) Non-Anemia group and the CTRL group, as well as shared features in (**C**) both disease groups compared to the CTRL group. Altered KO genes with a relative abundance of 0.1 or higher are shown. Significant changes (elevation and depletion) are denoted as follows: * q < 0.05, ** q < 0.01, *** q < 0.001, **** q < 0.0001.

In the Environmental Information Processing function, the phosphotransferase system (PTS) pathway genes (malX and gfrD), which are associated with the transport of carbohydrates, exhibited a significant downregulation in the IDA group compared with the CTRL group (Figure 4A and Table S7). Notably, the Non-Anemia group demonstrated an increase in ABC transporter genes (troA, mntA, znuA, troB, mntB, and znuC), which are responsible for bacterial iron acquisition (Figure 4B and Table S8). In the KO map of metabolism, the galactose metabolism pathway (genes agaA, lacE, lacF, and E3.2.1.85 involved) was significantly downregulated in the IDA group compared with that of the CTRL group (Figure 4A and Table S7). Studies showed that the intake of galactooligosaccharides enhanced iron absorption from ferrous fumarate [29]. In addition, megDIII, a gene participating in bacterial nucleotide sugar biosynthesis, was shown to increase exclusively in the IDA group (Figure 4A and Table S7). Meanwhile, the genes waaY and rfaY, related to bacterial wall polysaccharides and biofilm formation, as well as gene hdc, linked with histidine metabolism, were elevated in the Non-Anemia group (Figure 4B and Table S8). In the microbial metabolism in diverse environments, SCFA synthesis-related genes (acdA and lcdA) were depleted in the IDA group (Figure 4A and Table S7), and the methylaspartate synthesis-related genes (glmE, mutE, mamB, glmS, mutS, and mamA) were enriched in the Non-Anemia group (Figure 4B and Table S8). Furthermore, in the Cellular Processes pathways, the polysaccharide synthetic enzyme gene (pelG) was depleted in the IDA group (Figure 4A and Table S7), while protoporphyrin biosynthesis genes (PPOX and hemY) were elevated in the Non-Anemia group (Figure 4B and Table S8). The latter encoding protoporphyrinogen/coproporphyrinogen III oxidase is indispensable in the bacterial production of heme, the precursor of Hb [30].

In both disease groups, gene pdaD involved in arginine degeneration was upregulated, while genes belonging to the oxidative respiratory chain, exemplified by cypD_E, CYP102A, CYP505, CYTB, and petB, were downregulated (Figure 4C and Table S9). However, the common KO gene traits between the IDA group and the Non-Anemia group were virtually indistinguishable in their relative abundances (Table S9). Taken together, although the abundance of genes cannot fully predict the changes in microbial enzymatic reactions, the relative genes could steer the host’s iron utilization through the abnormalities in bacterial metabolisms.

### 3.6. Associations between the Disease-Linked Microbiota, Metabolites, and Relative KO Genes

**Figure 5:**
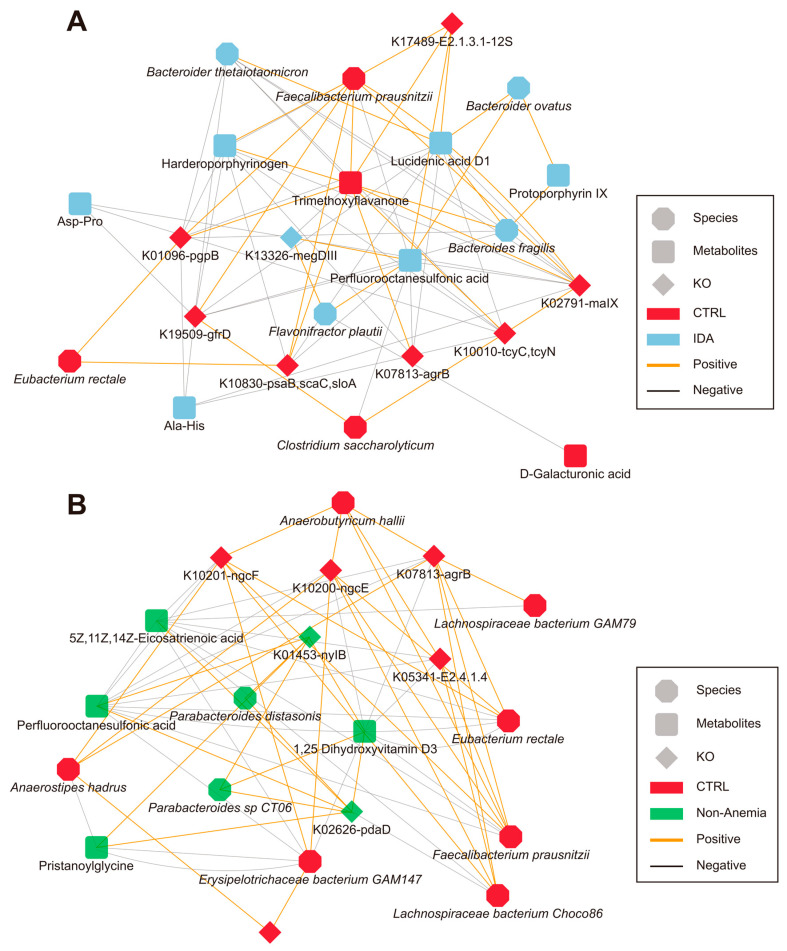
The correlation networks based on dual-omics in the IDA and Non-Anemia groups. The network reveals representatively significant associations (*p* < 0.05, Spearman rank correlation analysis) among differentially abundant taxa, metabolites, and KO genes (**A**) between 62 IDA and 244 CTRL patients, and (**B**) between 215 Non-Anemia and 244 CTRL patients, respectively. Nodes are colored according to the group in which features were enriched compared with the CTRL group. Lines connecting nodes indicate positive (orange) or negative (grey) correlations.

Our dual-omics data implicated the potential interactions among taxonomic, metabolic, and KO genetic signatures (Procrustes analysis, Table S10). Therefore, we performed correlation analyses to investigate the associations between species, metabolite profiles, and annotated KO genes in an attempt to depict unique microbial signatures of the IDA group and the Non-Anemia group in a network diagram. We observed strong positive associations between species and metabolites that were elevated in both disease groups, as well as negative associations between CTRL-enriched species and disease-enriched metabolites. Our results found that the reduced level of trimethoxyflavanone was closely related to the increased *F. plautii* and the nucleotide sugar biosynthesis-related KO gene megDIII in the IDA group. Notably, there was a strong positive correlation between disease-enriched lucidenic acid D1 and *Bacteroides fragilis* in the IDA group (Figure 5A and Table S11). Furthermore, 1,25-dihydroxyvitamin D3 was enriched in the Non-Anemia group and positively correlated with the Non-Anemia-specific taxa *Parabacteroides* sp. *CT06* and *Parabacteroides distasonis* and the KO gene nylB that encodes amide hydrolase. Elevated eicosatrienoic acid was negatively linked with the CTRL-enriched taxa *Lachnospiraceae bacterium, Choco86*, *Faecalibacterium prausnitzii,* and *Eubacterium rectale*, along with CTRL-enriched acetylglucosamine transporter-related KO genes ngcE and ngcF (Figure 5B and Table S11). As for features collectively possessed by both disease groups, the CTRL-enriched taxa, such as *Faecalibacterium prausnitzii* and *Eubacterium rectale*, were negatively associated with the increased perfluorooctanesulfonic acid yet positively linked with the decreased valeric acid. Moreover, the CTRL-depleted taxa *Bacteroides fragilis* was positively correlated with increased arginine metabolism-related KO gene pdaD (Table S11).

In addition, we also constructed random forest classifier (RFC) models that distinguished 61 patients with IDA and 215 non-anemia patients from 244 healthy control patients and used them to investigate the potential of gut microbial, metabolic, and KO gene profiles to predict IDA in CRC. The satisfactory performance of RFCs was also validated in the testing phase (Figure S4, Table S12). These three-feature integrated markers underlie promising non-invasive tools for the evaluation of iron-deficient status.

Conclusively, our data suggest that alterations in gut microbial metabolites and KO genes were associated with the changes in microbiota, and the combined analysis of the three types of data may partially explain the pathogenesis of the IDA condition in CRC patients and push for further explorations.

### 3.7. Microbial Prognostic Predictors of Cancer Recurrence

**Figure 6:**
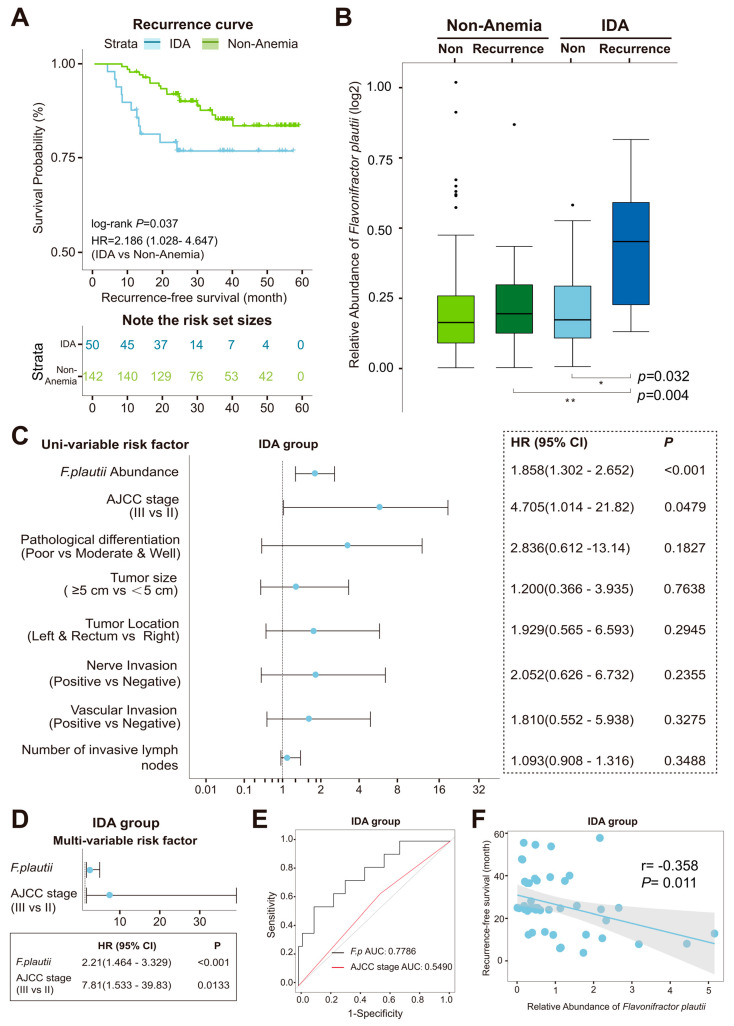
The association between *F. plautii* and cancer recurrence. (**A**) Kaplan–Meier curve for recurrence probability of the two groups and table of the number at risk analyzed by the Log-rank test (n = 192). (**B**) Statistical analysis of the relative abundance of *F. plautii* in the IDA and Non-Anemia groups using the Wilcoxon rank-sum test. * *p* < 0.05, ** *p* < 0.01. (**C**) Univariate analysis performed in the IDA group. The bars correspond to 95% confidence intervals. (**D**) Multivariate analysis performed in the IDA group. The bars correspond to 95% confidence intervals. (**E**) Receiver operating characteristic (ROC) analysis applied based on the relative abundance of *F. plautii* and the AJCC stage of CRC. (**F**) Linear correlation of RFS with the relative abundance of *F. plautii* in the IDA group.

Now that we have found an association between intestinal flora and its metabolism in CRC-associated IDA, we would like to further explore whether dysbiosis of intestinal flora affects the prognosis of patients with CRC-associated IDA. We collected information on the recurrence survival of 192 patients with stage II–III CRC. Kaplan–Meier survival analysis further intuitively unfolded the recurrence probability of the two disease groups. CRC patients with IDA were prone to undergoing cancer relapse with substantially shorter recurrence-free survival (RFS) (Figure 6A and Table S13). Based on the aforementioned result that the upward trend of *F. plautii* accompanied the progression of anemia, we hypothesized that the enrichment of *F. plautii* correlated with CRC recurrence and quantified the relative abundance of *F. plautii* in feces samples from 163 patients without recurrence and from 29 patients with recurrence. In the IDA group, the amount of *F. plautii* was significantly higher in recurrent patients than that in non-recurrent patients (Figure 6B and Table S13).

Furthermore, a univariate regression model of the IDA group was executed to estimate the performances of *F. plautii* abundance and standard clinicopathologic prognosticators, and a multivariate regression model of the IDA group revealed the potential value of *F. plautii* (HR = 2.21, 95% CI: 1.464–3.329) as a significant indicator of CRC poor outcomes comparable to those of the AJCC stage (HR = 7.81, 95% CI: 1.533–39.83) (Figure 6C,D and Table S14).

The AUC of *F. plautii* reached 0.78, recognizing that its performance in recurrence prediction was higher than that of the AJCC stage (AUC: 0.55) (Figure 6E). Linear correlation analysis showed that a higher relative abundance of *F. plautii* was significantly associated with a shorter RFS (r = −0.358, *p* = 0.011) (Figure 6F). The microbiota and metabolites distinctly altered in the IDA or Non-Anemia group also significantly achieved correlation coefficients towards RFS (Figure S5). Our results added to the evidence that the newly discovered microbial and metabolic signatures in anemic and non-anemic conditions could be leveraged as predictors of CRC recurrence.

## 4. Discussion

In this study, we conducted dual-omics analyses in IDA and Non-Anemia patients with CRC. The unique altered signatures were unveiled, and integrated analyses exposed exclusive reactions among taxa, metabolites, and KO genes, providing new insights into the mechanisms of two hematologic conditions from the perspective of the gut microbiome and metabolome.

Both the IDA group and the Non-Anemia group showed reduced α diversity and deviations in microbial communities compared with the CTRL group. The constantly altered taxa, metabolites, and relative KO genes were identified afterwards. *Faecalibacterium prausnitzii*, *Eubacterium rectale,* and *Roseburia intestinalis* were decreased in accordance with the results from previous feces sequencing in large CRC cohorts [31,32]. These bacteria and their fermented metabolites of SCFAs (including valeric acid) could potentially suppress chronic intestinal inflammation and maintain intestinal health [33]. A significant increase in perfluorooctanesulfonic acid (PFOS) was detected. Exposure to PFOS has been reported to result in notably higher risks of all-cause cancers compared to average levels [34].

By integrating dual-omics data, our study further revealed that the potentially oncogenic species *Bacteroides fragilis* was positively associated with the KO gene pdaD, highlighting an increase in arginine degeneration. Arginine-related pathways, including polyamines and proline synthesis, or the arginine/nitric oxide pathway, are reprogrammed in tumors and exert complex effects on the tumor microenvironment [35]. Our results infer that arginine is an important tumor suppressor, as previously published [36].

Although the fecal microbiomes and metabolomes of the IDA group and the Non-Anemia group overlapped in some characteristics when both were compared with the CTRL group, we still found unique microbial hallmarks in each CRC group. For example, the IDA group was characterized by elevated protoporphyrin IX. Iron deficiency results in large amounts of drifted protoporphyrin IX as tumors progress and bleed, which can be measured and aid in monitoring the degree of chronic hemorrhage [37]. Notably, the further increase in *F. plautii* from the non-anemic condition towards iron deficiency was negatively correlated with the decrease in trimethoxyflavanone, reflecting reduced intake or intensified degradation of flavonoids in CRC patients with IDA [38,39]. Flavonoids are polyphenolic compounds that can shield the positive charge of metal ions, thereby enhancing iron absorption by intestinal enterocytes [40]. Furthermore, accumulating evidence repeatedly supports the role of flavonoids and their secondary metabolites, polyphenols, in anti-inflammation [41,42]. Thus, decreased flavonoids are incapable of alleviating tumor-induced intestinal inflammation, which might in turn upregulate the expression of hepcidin (one of the “valves” regulating iron metabolism) via the IL6/STAT3 pathway, resulting in a limitation of bioavailable iron and ultimately a functional shortage of hematopoietic raw materials [43]. Flavonoids have been analogously proven to interfere with DNA/RNA synthesis, induce antimicrobial activities, and inhibit quorum sensing in bacteria [44]. Our analyses revealed that the elevation of megDIII, a KO gene that is involved in the biosynthesis of nucleotide sugars, was associated with the decrease in trimethoxyflavanone and the increase in *F. plautii*, implying an accelerated growth of the pathogen fueled by the final products (including ATP) of trimethoxyflavanone after complex reactions. In conclusion, we explored the contribution of chronic bleeding and a mounting inflammatory tendency to host iron dyshomeostasis, which warrants further investigation.

In a non-anemic environment with relative iron sufficiency, genes related to the iron transport system along with heme biosynthesis are comparatively upregulated, which is a manifestation of intense competition with tumor cells over gut luminal iron [45,46]. Moreover, the significant upregulation of *Parabacteroides* sp. *CT06* and *Parabacteroides distasonis* observed only in the Non-Anemia group was positively correlated with the increased 1,25-dihydroxyvitamin D3. Prior evidence has shown that a 1,25-dihydroxyvitamin D3 supplement altered microbiota composition by increasing the relative abundance of a series of bacteria, including Parabacteroides [47], among which *Parabacteroides distasonis* is competent to metabolize bile acids to produce lithocholic acid (LCA). LCA is a strong agonist of the vitamin D receptor (VDR) [48]; thus, the activation of VDR signaling contributes to the maintenance of gut barrier integrity and the regulation of pro-inflammatory factors [49,50]. This pathway remains to be validated in the non-anemic scenario of CRC patients. More importantly, vitamin D has also been proven to inhibit hepcidin expression directly via VDR signaling and further release restricted iron by upregulating ferritin’s iron export activity, paving the way for subsequent Hb synthesis and thus restoring hematological parameters. The rewiring of iron metabolism in the gut of non-anemic CRC patients mainly emphasizes the vigorous VDR signaling stimulated by the metabolites of Parabacteroides and 1,25-dihydroxyvitamin D3. These findings implied that the alleviation of inflammatory conditions helped disrupt iron metabolism.

Several studies have reported that some of the potentially oncogenic microorganisms of the intestinal tract are valuable as markers for monitoring the prognosis of CRC [51]. We further discovered that CRC patients in the IDA group with a higher abundance of *F. plautii* in the gut faced a notably elevated susceptibility to recurrence and identified potential relationships between anemic microbial signatures and RFS. The mechanisms by which *F. plautii* accounts for worse oncological outcomes are poorly understood. Adding the relative abundance of *F. plautii* to current predictive models, which are mainly based on the heterogeneity of tumors and characteristics of patients [52,53,54], might increase the sensitivity and specificity of recurrence prediction and accurately select the target population that tends to benefit from adjuvant therapies.

Several limitations have to be acknowledged in our study. First, this was a single-center study that was not validated with external cohorts from multiple regions, which may weaken the generalizability and adaptability of the findings. Second, although we adjusted covariates including age and gender in our analyses, there were other confounders, including dietary habits and physiological characteristics of the hosts. A third shortcoming is that our study did not demonstrate the endoscopic manifestations of colorectal tumors, e.g., fibro-colonoscopic tumor morphology in IDA patients with gut bleeding and fibro-colonoscopic tumor morphology in non-anemic patients with gut non-bleeding. An attempt will be made to validate the robustness of the diagnostic model against larger samples and clinical data to dissect the causal relationship between fecal bacteria, their metabolites, and IDA.

**Figure 7:**
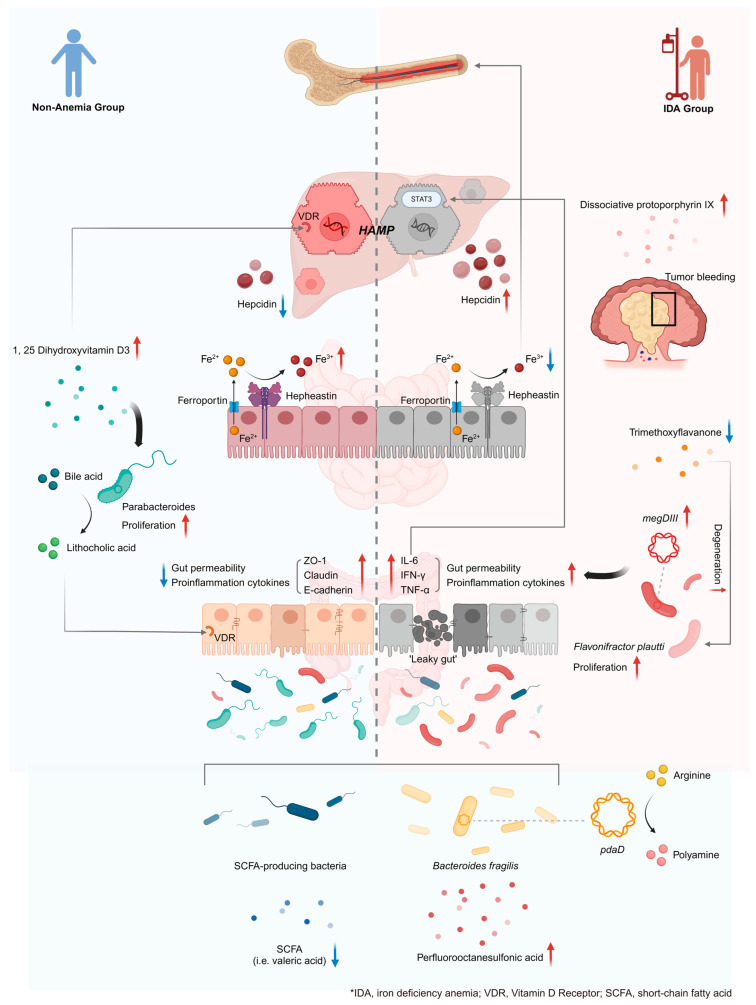
The landscape of gut bacteria, metabolites, and KO genes in the IDA and Non-Anemia patients. The Non-Anemia group is characterized by elevated VDR signaling activated by the metabolites of Parabacteroides and 1,25-dihydroxyvitamin D3, leading to the downregulation of the hepcidin-ferroportin axis, which liberates sequestered iron and promotes erythropoiesis (left hemi-figure). The three-feature profile of the IDA group depicts that enrichment of *F. plautii*, downregulation of the KO gene megDIII related to bacterial proliferation, and decreased trimethoxyflavanone jointly become involved in the ‘leaky gut’ and overexpression of hepcidin. It is also worth noting that the elevated protoporphyrin IX presents the view that the constant tumor bleeding may be the culprit of IDA (right hemi-figure). Shown at the bottom are overlapping signatures associated with CRC, including reductions in SCFA-producing bacteria and corresponding valeric acid depletion. *, abbreviations; up arrows, increased; down arrows, decreased.

In summary, the present study is an encouraging start to developing a dual-omics research paradigm in which we have identified critical alterations in gut microbiome species, metabolites, and KO genes that largely reflect the symptoms of iron deficiency in patients with CRC (Figure 7). More exacting studies need to be conducted to testify to the posed assumption in our finding concerning the mechanisms of the development of CRC-related IDA and the unsatisfactory survival of CRC-related IDA patients, underpinning the knowledge of assessment of fecal microbial outcomes as a non-invasive test to guide the curative paradigm of IDA derived from CRC.

## Figures and Tables

**Table 1:** Clinical characteristics of the enrolled participants in the cohort.

Characteristics	CTRL n = 244	Non-Anemia n = 245	IDA n = 69	*p* Value (CTRL vs. Non-Anemia)	*p* Value (CTRL vs. IDA)	*p* Value (Non Anemia vs. IDA)
Age, year, mean (±SD)	49.94 (13.56)	54.10 (13.23)	50.80 (14.69)	0.559	0.485	0.272
Gender, N (%)						
Male	107 (43.8)	153 (62.4)	25 (36.2)	0.156	0.594	0.104
Female	137 (56.1)	92 (37.5)	44 (63.7)			
BMI (kg/m^2^), N (%)				<0.0001	<0.0001	0.053
<24	86 (35.2)	135 (55.1)	47 (68.1)			
≥24	158 (64.8)	110 (44.9)	22 (31.9)			
Hemoglobin level (g/L)						
Male Hb, mean (±SD)	NA	145.32 (15.33)	98.68 (18.76)			0.014
Female Hb, mean (±SD)	NA	131.69 (14.42)	88.45 (15.67)			0.041
Hematocrit (%)						
Male HCT, mean (±SD)	NA	43.23 (2.72)	32.04 (5.12)			<0.0001
Female HCT, mean (±SD)	NA	39.49 (2.67)	29.20 (4.44)			<0.0001
Tumor location, N (%)				<0.0001	<0.0001	<0.0001
Right hemicolon	NA	34 (13.9)	39 (39.0)			
Left hemicolon	NA	73 (29.8)	24 (24.0)			
Rectum	NA	138 (56.3)	37 (37.0)			
Tumor size, maximum diameter (cm), N (%)						0.020
<5	NA	187 (76.3)	43 (62.3)			
≥5	NA	58 (23.7)	26 (37.7)			
Differentiation, N (%)						0.558
Well-moderate	NA	174 (71.3)	46 (67.6)			
Poor	NA	70 (28.7)	22 (32.4)			
TNM stage, N (%)						0.421
I-II	NA	123 (50.4)	31 (44.9)			
III-IV	NA	121 (49.6)	38 (55.0)			
Non-quantitative FOBT, N (%)						0.520
Negative	NA	142 (58.0)	37 (53.6)			
Positive	NA	103 (42.0)	32 (46.3)			
Albumin (g/L), N (%)						<0.0001
<40	NA	21 (8.6)	22 (31.9)			
≥40	NA	205 (83.7)	47 (68.1)			
Prealbumin (mg/L), N (%)						<0.0001
<280	NA	152 (67.6)	61 (91.0)			
≥280	NA	73 (32.4)	6 (9.0)			
CEA (mg/L), N (%)						0.160
≤5.9	NA	169 (69.8)	42 (60.9)			
>5.9	NA	73 (30.2)	27 (39.1)			
Nerve invasion, N (%)						0.837
Negative	NA	172 (70.8)	49 (72.1)			
Positive	NA	71 (29.2)	19 (27.9)			
Vascular invasion, N (%)						
Negative	NA	155 (63.5)	37 (54.5)			0.172
Positive	NA	89 (36.5)	31 (46.5)			

Abbreviations: BMI, body mass index; NA, not available; Hb, hemoglobin; HCT, hematocrit; FOBT, fecal occult blood test; CEA, carcinoembryonic antigen.

## Data Availability

The raw sequence data reported in this paper have been deposited in the Genome Sequence Archive in the National Genomics Data Center, China National Center for Bioinformation/Beijing Institute of Genomics, Chinese Academy of Sciences (GSA-Human: HRA005038), which is publicly accessible at https://ngdc.cncb.ac.cn/gsa-human. The raw metabolomics data have been deposited in the OMIX, China National Center for Bioinformation/Beijing Institute of Genomics, Chinese Academy of Sciences (https://ngdc.cncb.ac.cn/omix/view/OMIX004606). Any additional information required to reanalyze the data reported in this paper is available from the lead contact upon request.

## Supplementary Materials

The following supporting information can be downloaded at: https://www.mdpi.com/article/10.3390/microorganisms12071319/s1, Figure S1: Fecal microbiome variations. (A) Alpha diversity index measured by the Chao1 Index. (B) Beta diversity and principal coordinate analysis. Figure S2: The distribution of metabolites and KO genes. (A) The distribution of metabolites in the PLS-DA analysis. (B) The distribution of KO genes in the PCoA analysis. Figure S3: Venn diagram of the metabolic signatures. Figure S4: Integrative three-feature markers of the IDA and Non-Anemia groups by random forest models. Figure S5: The correlations of RFS with relative abundances of features in the IDA and Non-Anemia groups. (A) Linear correlation of RFS with a relative abundance of prostaglandin H2 in the IDA group. (B) Linear correlation of RFS with a relative abundance of *Parabacteroides distasonis* in the Non-Anemia group. (C) Linear correlation of RFS with a relative abundance of L-Histidine in the Non-Anemia group. Table S1: The abundance of bacterial species differed between the IDA and CTRL groups. Table S2: The abundance of bacterial species differed between the non-anaemic and CTRL groups. Table S3: Comparison of the abundance of bacterial species in the IDA and Non-Anemia groups. Table S4: Differences in fecal metabolites between the IDA and CTRL groups. Table S5: Differences in fecal metabolites between the Non-Anemia and CTRL groups. Table S6: Differences in fecal metabolites between the IDA and Non-Anemia groups. Table S7: Differences in the KO genes between the IDA and CTRL groups. Table S8: Differences in the KO genes between the Non-Anemia and CTRL groups. Table S9: Differences in the KO genes between the IDA and Non-Anemia groups. Table S10: The associations between taxa, metabolites, and annotated KO genes using Procrustes analysis. Table S11: The list of significant associations among taxa, metabolites, and KO genes. Table S12: The performances of all prediction models. Table S13: Information on tumor recurrence. Table S14: The multivariable risk factors for recurrence in the IDA group.
