# Supplementary material for: Characteristics of Gut Microbiota and Fecal Metabolites in Patients with Colorectal Cancer-Associated Iron Deficiency Anemia
Source: Microorganisms. 2024 Jun 28;12(7):1319. doi: 10.3390/microorganisms12071319 (PMC11279063; doi:10.3390/microorganisms12071319)
Supplement: Supplementary file 1 [file microorganisms-12-01319-s001.zip › Figure_S3.pdf]

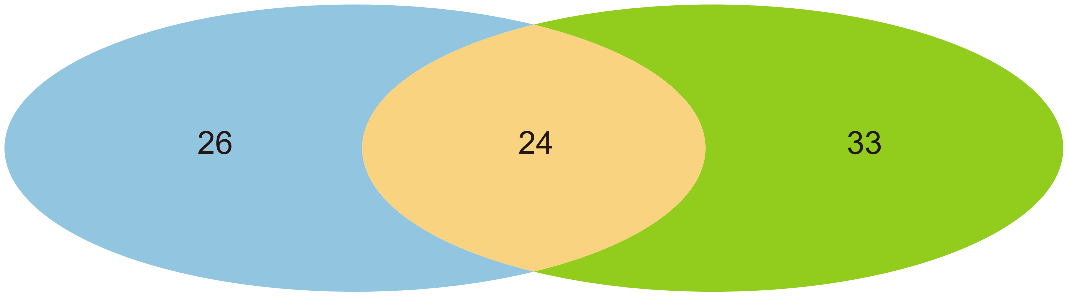

A: IDA signature

Trimethoxyflavanone
Protoporphyrin IX
Prostaglandin
D-Galacturonic acid
Austroinulin
Quinic acid

C: CRC signature

Perfluorooctanesulfonic acid
Valeric acid
N-Oleoyl phenylalanine
2-Indolecarboxylic acid

B: Non-Anemia signature

1,25 Dihydroxyvitamin D3
5Z,11Z,14Z-Eicosatrienoic acid
N-Stearoyl GABA
Phosphatidylcholine
L-Histidine
Phe-Pro
Acetyl-DL-valine
Pristanoylglycine
