# Supplementary material for: Characteristics of Gut Microbiota and Fecal Metabolites in Patients with Colorectal Cancer-Associated Iron Deficiency Anemia
Source: Microorganisms. 2024 Jun 28;12(7):1319. doi: 10.3390/microorganisms12071319 (PMC11279063; doi:10.3390/microorganisms12071319)
Supplement: Supplementary file 1 [file microorganisms-12-01319-s001.zip › Figure_S4.pdf]

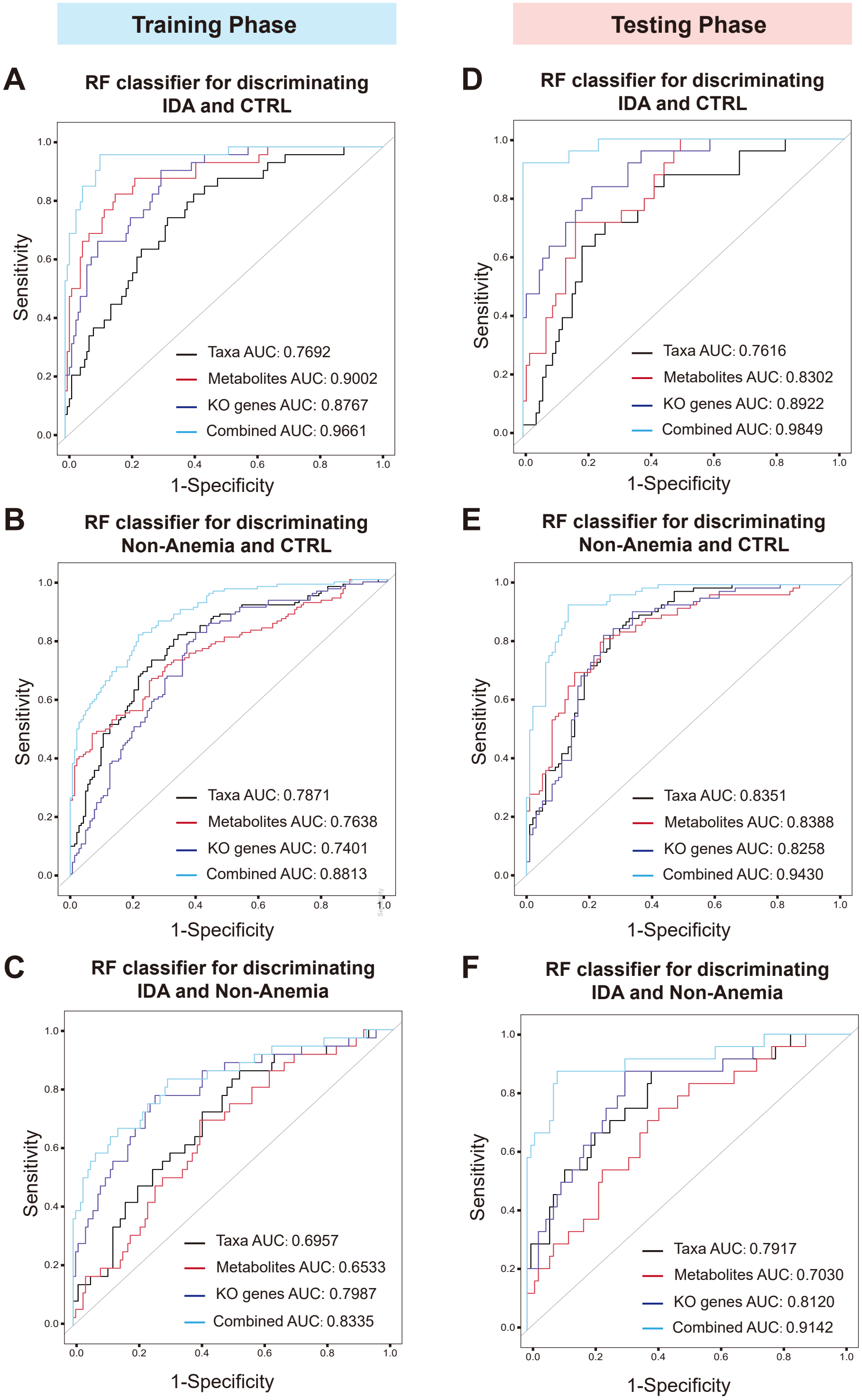
Training Phase
Testing Phase
A
RF classifier for discriminating
IDA and CTRL
Sensitivity
1-Specificity
Taxa AUC: 0.7692
Metabolites AUC: 0.9002
KO genes AUC: 0.8767
Combined AUC: 0.9661
D
RF classifier for discriminating
IDA and CTRL
Taxa AUC: 0.7616
Metabolites AUC: 0.8302
KO genes AUC: 0.8922
Combined AUC: 0.9849
B
RF classifier for discriminating
Non-Anemia and CTRL
Taxa AUC: 0.7871
Metabolites AUC: 0.7638
KO genes AUC: 0.7401
Combined AUC: 0.8813
E
RF classifier for discriminating
Non-Anemia and CTRL
Taxa AUC: 0.8351
Metabolites AUC: 0.8388
KO genes AUC: 0.8258
Combined AUC: 0.9430
C
RF classifier for discriminating
IDA and Non-Anemia
Taxa AUC: 0.6957
Metabolites AUC: 0.6533
KO genes AUC: 0.7987
Combined AUC: 0.8335
F
RF classifier for discriminating
IDA and Non-Anemia
Taxa AUC: 0.7917
Metabolites AUC: 0.7030
KO genes AUC: 0.8120
Combined AUC: 0.9142
