# Supplementary figures and images for: Characteristics of Gut Microbiota and Fecal Metabolites in Patients with Colorectal Cancer-Associated Iron Deficiency Anemia
Source: Microorganisms. 2024 Jun 28;12(7):1319. doi: 10.3390/microorganisms12071319 (PMC11279063; doi:10.3390/microorganisms12071319)

**A**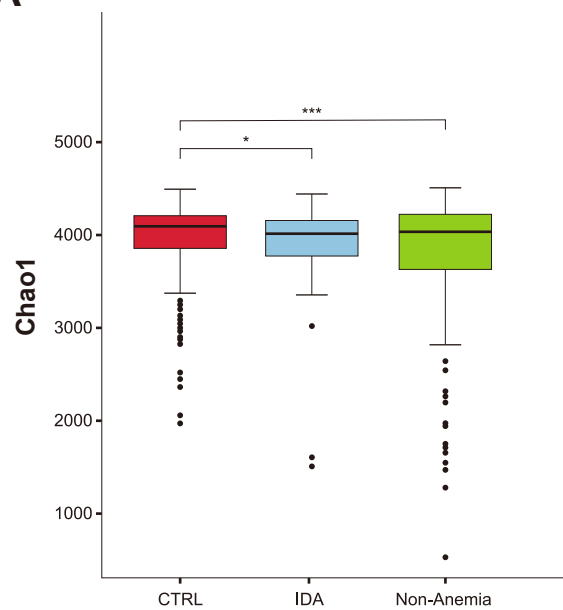**B**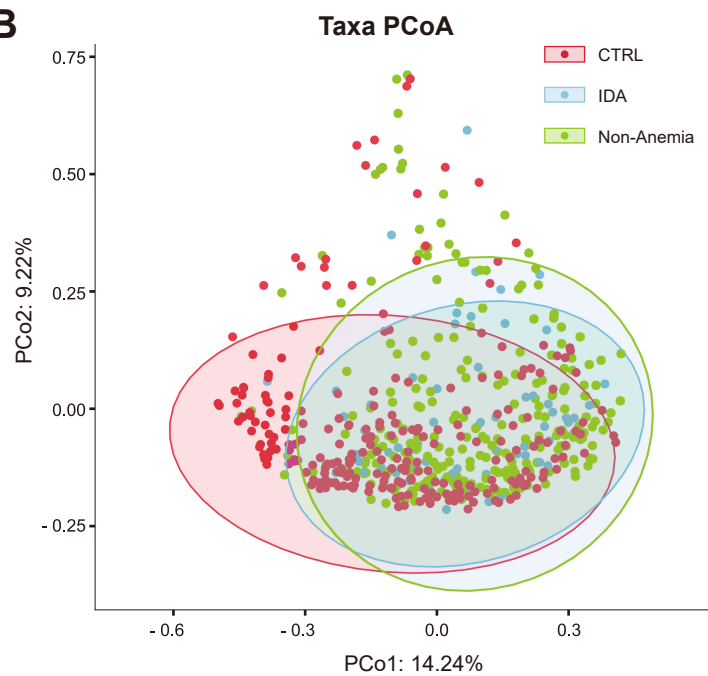

Supplement: Supplementary file 1 [file microorganisms-12-01319-s001.zip › Figure_S1.pdf]

**A****Metabolites PLS-DA**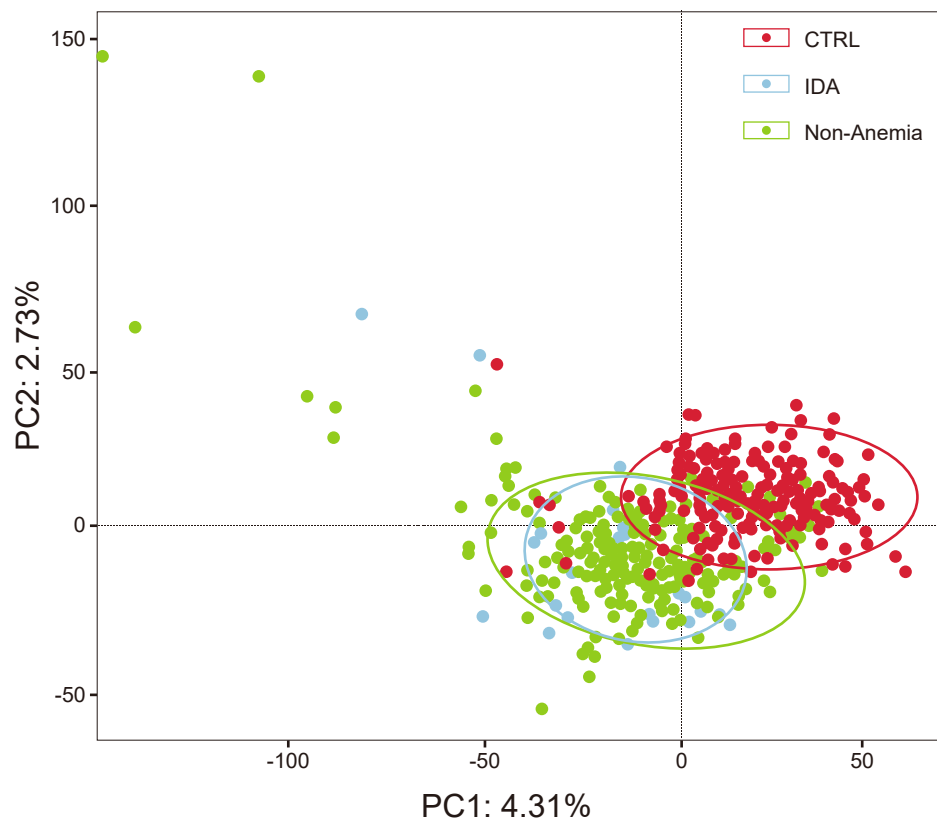**B****KO PCoA**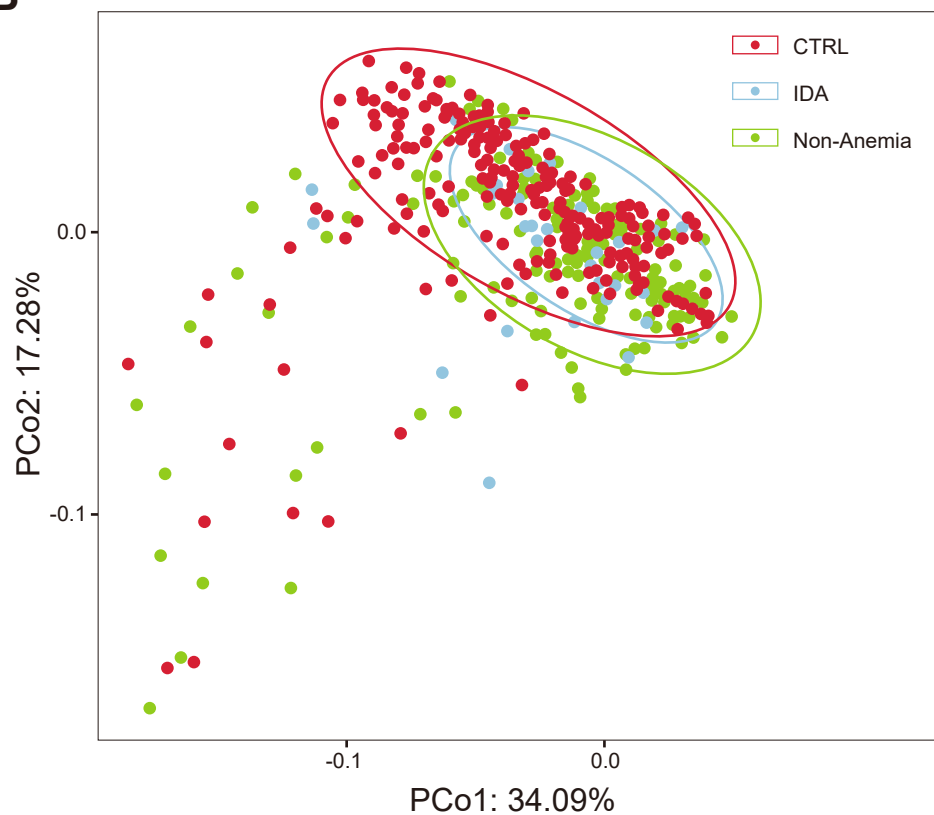

Supplement: Supplementary file 1 [file microorganisms-12-01319-s001.zip › Figure_S2.pdf]

### A IDA metabolic signature

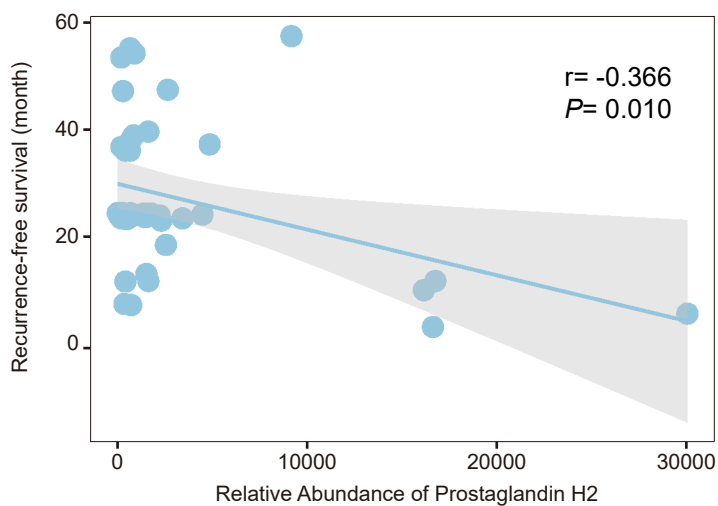

### B Non-Anemia bacterial signature

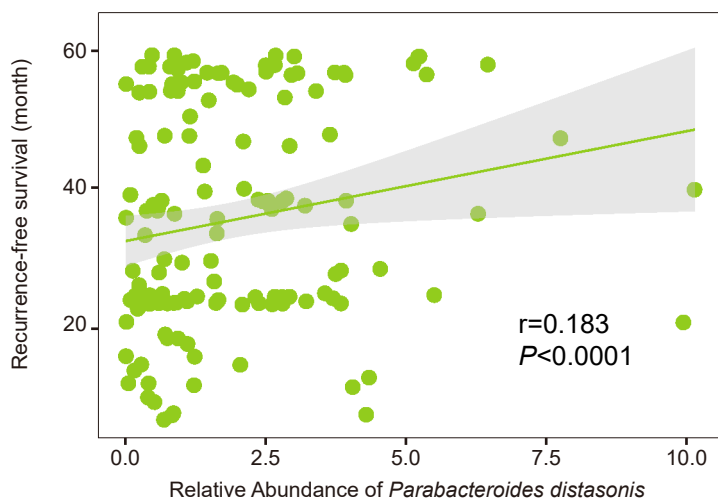

### C Non-Anemia metabolic signature

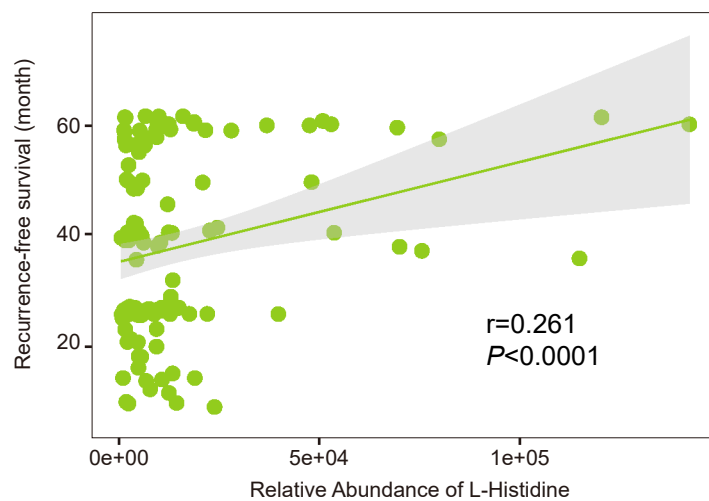

Supplement: Supplementary file 1 [file microorganisms-12-01319-s001.zip › Figure_S5.pdf]
